# Supplementary material for: Differential LRRK2 Signalling and Gene Expression in WT-LRRK2 and G2019S-LRRK2 Mouse Microglia Treated with Zymosan and MLi2
Source: Cells. 2023 Dec 26;13(1):53. doi: 10.3390/cells13010053 (PMC10778119; doi:10.3390/cells13010053)
Supplement: Supplementary file 1 [file cells-13-00053-s001.zip › 3. Supplementary figure S1_IN 14-11 edits.pptx]

## Slide 1
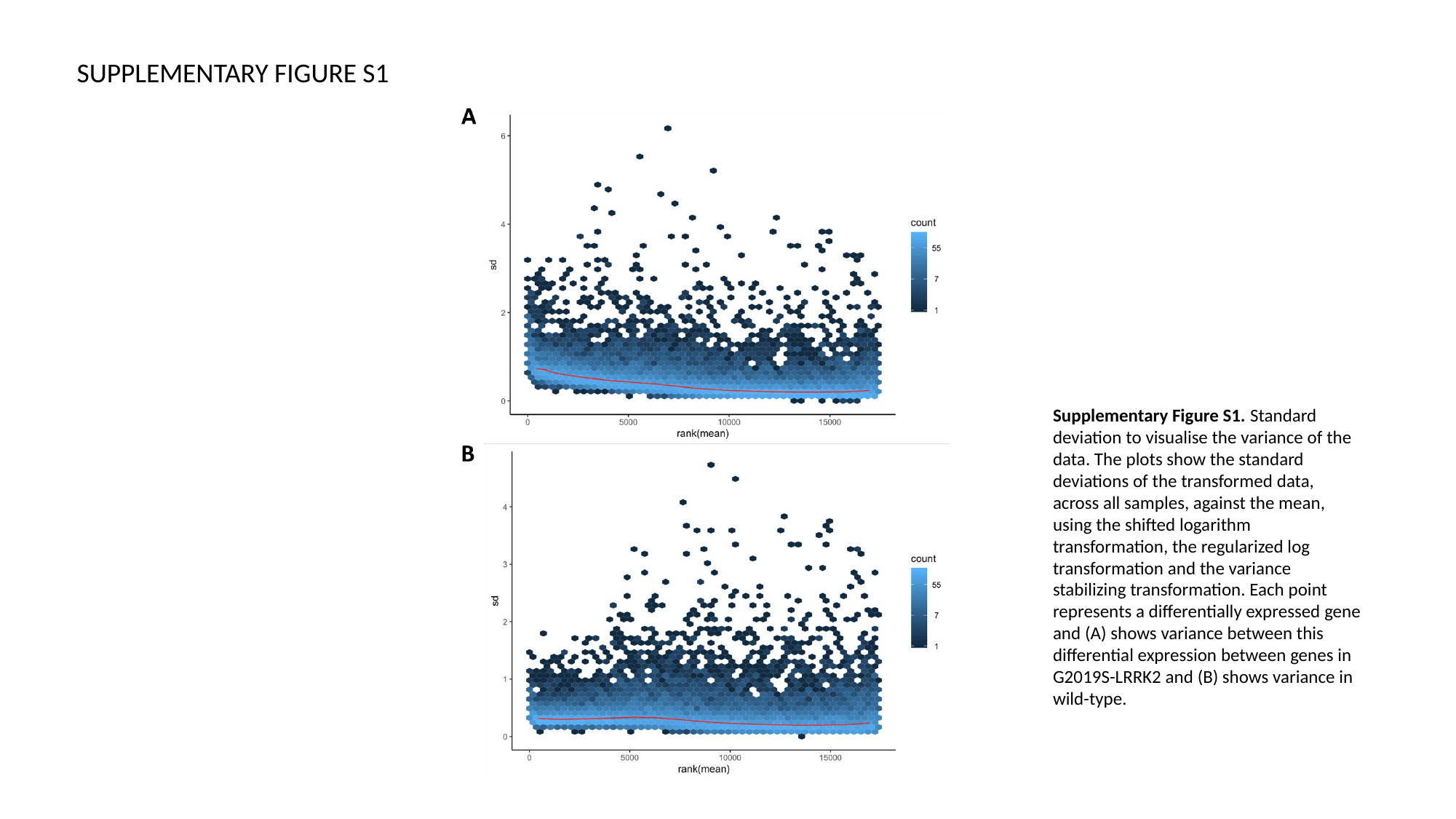

SUPPLEMENTARY FIGURE S1
Supplementary Figure S1. Standard deviation to visualise the variance of the data. The plots show the standard deviations of the transformed data, across all samples, against the mean, using the shifted logarithm transformation, the regularized log transformation and the variance stabilizing transformation. Each point represents a differentially expressed gene and (A) shows variance between this differential expression between genes in G2019S-LRRK2 and (B) shows variance in wild-type.

## Slide 2
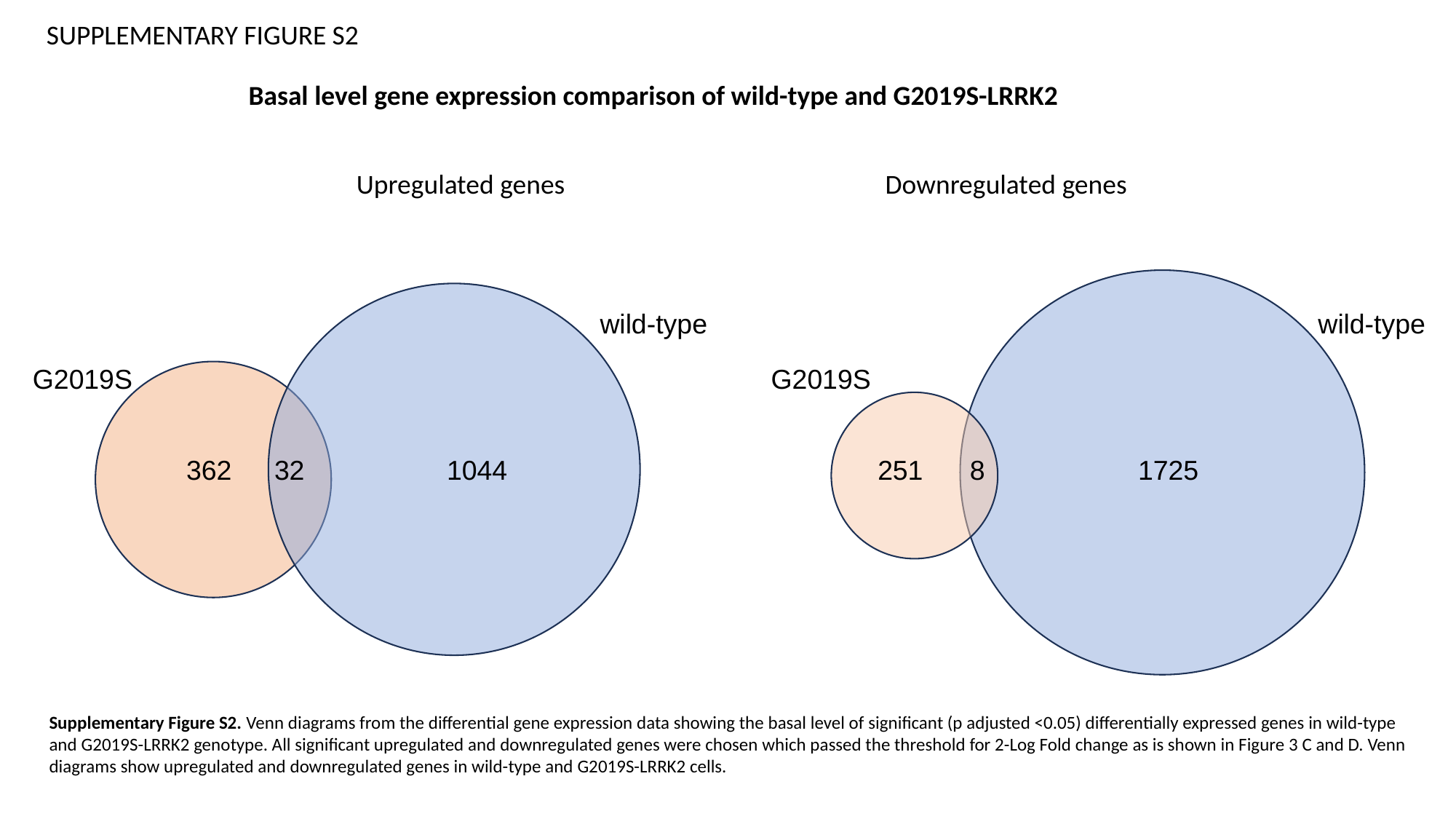

SUPPLEMENTARY FIGURE S2
Basal level gene expression comparison of wild-type and G2019S-LRRK2
Upregulated genes
Downregulated genes
wild-type
wild-type
G2019S
G2019S
362
32
1044
251
8
1725
Supplementary Figure S2. Venn diagrams from the differential gene expression data showing the basal level of significant (p adjusted <0.05) differentially expressed genes in wild-type and G2019S-LRRK2 genotype. All significant upregulated and downregulated genes were chosen which passed the threshold for 2-Log Fold change as is shown in Figure 3 C and D. Venn diagrams show upregulated and downregulated genes in wild-type and G2019S-LRRK2 cells.
